# PASSerRank: Prediction of Allosteric Sites with Learning to Rank

**Published:** 2023-02-02

**Authors:** Hao Tian, Sian Xiao, Xi Jiang, Peng Tao

**Affiliations:** † Department of Chemistry, Center for Research Computing, Center for Drug Discovery, Design, and Delivery (CD4), Southern Methodist University, Dallas, Texas, United States of America; ‡ Department of Statistical Science, Southern Methodist University, Dallas, Texas, United States of America

## Abstract

Allostery plays a crucial role in regulating protein activity, making it a highly sought-after target in drug development. One of the major challenges in allosteric drug research is the identification of allosteric sites, which is a fundamental aspect of the field. In recent years, many computational models have been developed for accurate allosteric site prediction. However, most of these models focus on designing a general rule that can be applied to pockets of proteins from various families. In this study, we present a new approach to this task using the concept of Learning to Rank (LTR). Our LTR model ranks pockets of each protein based on their relevance as allosteric sites. The model outperforms other common machine learning models, with a higher F1 score and Matthews correlation coefficient. After training and validation on two datasets, the Allosteric Database (ASD) and CASBench, the LTR model was able to rank an allosteric pocket in the top 3 positions for 83.6% and 80.5% of test proteins, respectively. The trained model is available for free on the PASSer platform (https://passer.smu.edu) to aid in drug discovery research.

## 1 Introduction

Allostery is a biological process where an effector molecule binds to a site that is separate from the active site of a protein. This binding results in conformational and dynamical changes that can regulate the protein’s function, making it a key aspect of cellular signaling and considered as the second secret of life.^1–4^ Despite its importance, the allosteric mechanisms of most proteins remain unknown, with no universal mechanism having been discovered yet.^5^

Allostery offers several advantages in drug development. Compared to binding at the orthosteric site, allosteric site binding provides a controlled regulation of protein function that can either enhance or reduce the binding of ligands at the orthosteric site.^6^ Additionally, allosteric modulators are reported to have fewer side effects and no additional pharmacological effects once allosteric sites are fully occupied.^7^ Furthermore, allosteric sites experience low evolutionary pressure, ensuring the safety of on-target drugs.^8,9^ These benefits make allosteric drug development a promising field and offer substantial advantages over orthosteric drug development.

Identifying appropriate allosteric sites is a major challenge in allosteric drug development.^10,11^ In recent years, numerous computational methods for allosteric site identification and prediction have been developed. With the help of machine learning (ML), Allosite^12^ applies support vector machine (SVM) to learn the physical and chemical features of protein pockets. Another ML-based approach, the three-way random forest (RF) model developed by Chen *et al.*,^13^ is capable of predicting allosteric, regular, or orthosteric sites. PASSer^14,15^ is a recently developed method that combines extreme gradient boosting (XGBoost)^16^ with a graph convolutional neural network^17^ to learn physical and topological properties without any prior information. In addition to ML, traditional methods such as normal mode analysis (NMA)^18^ and molecular dynamics (MD)^19^ are widely used to investigate the communication between regulatory and functional sites, including SPACER^20^ and PARS.^21^ It is also important to note the development of allostery databases, including the Allosteric Database (ASD),^22^ which contains 1949 entries of protein-modulator complexes with annotated allosteric residues, and ASBench,^23^ a smaller benchmark set optimized from the ASD data. CASBench^24^ is a benchmarking set that includes annotated catalytic and allosteric sites. These datasets play a crucial role in training and evaluating allosteric site prediction models.

Most previous research on prediction models has focused on developing universal models for allosteric site prediction. These models intend to make ‘absolute’ predictions (either as labels or probabilities) for all pockets detected in different types of proteins, which is a challenging and time-consuming task. Learning to Rank (LTR), an emerging area, was first applied in information retrieval^25^ and has been used in many bioinformatics studies, ranging from drug-target interaction prediction^26^ to compound virtual screening.^27^ Unlike ‘absolute’ predictions, LTR models provide ‘relative’ predictions by ranking objects from the most to the least relevant, making it a more achievable and reasonable approach for the allosteric site prediction task.

In this study, we present the state-of-the-art machine learning model on allosteric site prediction using LambdaMART. LambdaMART model is implemented with gradient boosting decision tree (GBDT) and lambdarank loss function. Compared with other machine learning models such as XGBoost, SVM, and RF, LambdaMART achieved the highest F1 score and Matthews correlation coefficient (MCC). Moreover, this model has a better ability to rank actual allosteric sites at top positions. The trained LambdaMART model is freely available at PASSer (https://passer.smu.edu) to facilitate related research.

## 2 Methods

### 2.1 Allosteric Protein Databases

Two databases were used to train and validate different machine learning models, including the Allosteric Database (ASD) and CASBench.

In the latest version of ASD, there are 1949 entries of protein-modulator complexes. To ensure data quality, a filtration process is applied to the protein-modulator complexes based on standards proposed in the Allosite study.^12^ These standards include the requirement for high resolution protein structures with a resolution smaller than 3 *Å*, the presence of a complete structure in the allosteric site, and a low sequence identity threshold of 30% or greater. To facilitate the filtration process, a data processing pipeline script has been created and made available as open source on GitHub (https://github.com/smu-tao-group/PASSerRank).

The CASBench benchmark set comprises proteins annotated with allosteric sites, but only those entries that include both allosteric ligands and sites were included in the set. Additionally, proteins that were already present in the ASD dataset were removed to ensure the diversity of the benchmark set.

### 2.2 Pocket Descriptors and Labeling

FPocket is an open-source software for protein pocket detection. In this work, FPocket was applied on each protein to detect protein pockets. For each detected pocket, 19 physical and chemical features are calculated, ranging from pocket volume, solvent accessible surface area to hydrophobicity. A complete list of feature names is shown in Figure 2.

To label each pocket as an allosteric or non-allosteric site, we have automated the process by assigning the closest pockets to the modulator as the allosteric site. The center of mass is first calculated for all pockets and the modulator, and then the pairwise distances between the pockets and the modulator are computed. The pocket with the shortest distance is labeled as positive (allosteric site), while all other pockets are labeled as negative (non-allosteric site). However, if the closest distance is greater than 10 *Å*, this entry is removed from the dataset, as such a large distance may indicate inaccurate pocket detection and negatively impact the performance of the model.

### 2.3 Learning to Rank

Prior research on allosteric site prediction focus on developing a universal model that can accurately predict allosteric sites in all proteins. However, in practice, it is more important to identify the most promising pockets within each individual protein. Therefore, a machine learning model that is capable of ranking pockets in order of their likelihood to be allosteric sites is more desirable and attainable than a binary classification model that provides absolute predictions for all pockets.

In this study, we implement the LTR algorithm using gradient boosting decision tree (GBDT) and the LambdaMART method. GBDT is a popular machine learning approach that iteratively learns from decision trees and ensembles of their predictions. Here, we use LightGBM,^28^ one of the two popular implementations of GBDT, over XGBoost.^16^ LambdaMART is an LTR method that trains GBDT with the lambdarank loss function. The lambdarank loss function optimizes the value of the normalized discounted cumulative gain (NDCG) for the top *K* cases, and is calculated using discounted cumulative gain (DCG) and ideal discounted cumulative gain (IDCG) as:

(1) $$\text{DCG}@K=\sum_{i=1}^{K}\frac{2^{G_{i}}\;-\;1}{\log_{2}(i\;+\;1)}$$

(2) $$\text{IDCG}@\text{K=}\sum_{i=1}^{K}\frac{{\text{2}}^{\text{|}G{\text{|}}_{i}}\;-\;1}{\log_{\text{2}}(i\;+\;1)}$$

(3) $$\text{NDCG}@K\text{=}\frac{\text{DCG}@K}{\text{IDCG}@K}$$

where *G*_*i*_ is the gain (graded relevance value) at position *i* and |*G*| is the ideal ranking.

The LGBMRanker module in the LightGBM package (v3.3.4) was used to implementate the LambdaMART algorithm with GBDT as boosting type and lambdarank as the objective function.

### 2.4 Machine Learning Models

In addition to the LTR model, other commonly used machine learning models in allosteric site prediction were considered for comparison. XGBoost and RF are tree-based models. As previously stated, XGBoost is an implementation of the GBDT model that could also be uesd to train the LTR model. The RF model employs a bagging approach, training several independent decision trees in parallel. The prediction of RF is obtained through the weighted average of the outputs of all decision trees. The SVM classifier, on the other hand, learns a high-dimensional hyperplane that separates data points based on their labels. The XGBoost algorithm was implemented using the XGBoost package (version 1.7.3), and the RF and SVM classifiers were implemented using the Scikit-learn package (version 1.2.0).^29^

SHapley Additive exPlanations (SHAP) value is a method to increase model interpretability by quantifying feature importance. It has been implemented recently to explain tree-based models.^30^ In this study, the SHAP values of 19 features from FPocket were calculated and compared. The method is implemented in the SHAP^31^ package (v0.41.0).

### 2.5 Performance Criteria

Several metrics are calculated to compare and evaluate different machine learning models. Precision, recall, and specificity are good indicators for binary classification. The F1 score is a weighted measure of precision and recall. Moreover, it is reported that the Matthews correlation coefficient is a more suitable indicator than the F1 score and accuracy in binary classification evaluation.^32^

(4) Precision=TP/(TP+FP)

(5) Recall=TP/(TP+FN)

(6) Specificity=TN/(TN+FP)

(7) F1score=2*Precision*Recall/(Precision+Recall)

(8) Accuracy=(TP+TN)/(TP+FP+FN+TN)

(9) $$\text{MCC}=\frac{\text{TP}\;*\;\text{TN}\;-\;\text{FP}\;*\;\text{FN}}{\sqrt{\left(\text{TP}\;+\;\text{FP})\;*\;(\text{TP}\;+\;\text{FN})\;*\;(\text{TN}\;+\;\text{FP})\;*\;(\text{TN}\;+\;\text{FN}\right)}}$$

In order to evaluate the performance of the models, the percentage of actual allosteric sites that are ranked in the top 1, 2, and 3 positions is calculated. This metric is commonly used in evaluating various allosteric site prediction models. The actual allosteric sites are compared with the predicted top 3 most probable pockets in each protein, and the percentage is calculated and accumulated for each position.

## 3 Results

In this study, we adhered to three established standards and the pocket labeling strategy while preparing the training data for machine learning models. To ensure the quality of the protein-modulator complexes, we only considered those with high resolution protein structures (i.e., with a resolution of less than 3*Å*) as reported in the RCSB Protein Data Bank.^33^ Any protein structures that were missing modulators were excluded from the analysis.

**Figure 1.**
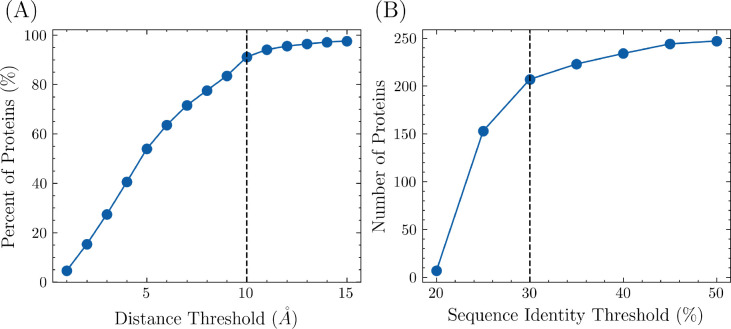
The number of proteins included in the training set, along with different distance and sequence identity thresholds. (A) The minimum distances between the center of masses from pockets to the modulator in each protein were calculated. One protein-modulator complex is discarded if the minimum distance is higher than the threshold. With the threshold being set as 10 *Å*, 91.1% of proteins were included. (B) To ensure uniqueness, proteins with high sequence identity were removed. The threshold was set as 30% to include 207 proteins in the training set.

FPocket was initially used to identify potential pockets in each protein, after which the center of mass was calculated for both the pockets and the modulator. The pairwise distances between the center of mass of each pocket and the modulator were calculated, and the closest pocket to the modulator was designated as the allosteric site while the other pockets were designated as non-allosteric sites. In order to ensure that the allosteric site and modulator were in contact, a distance threshold was imposed on the closest pocket. The effect of different distance thresholds on the percent of proteins included in the training set is shown in Figure 1(A), with a final distance threshold of 10 *Å* chosen to avoid the inclusion of incorrectly labeled pockets. Consequently, 91.1% of proteins from the ASD were included in the training set. To avoid overrepresentation of highly similar proteins, the pairwise sequence similarity was calculated between each newly selected protein and all previously selected proteins. If the similarity was higher than a specified threshold, the protein structure was discarded. The effect of different sequence identity thresholds is shown in Figure 1(B), with a final threshold of 30% chosen. After these steps, 207 proteins were included in the overall training set.

We randomly selected 80% of these proteins as the training set and used the remaining 20% as testing set. A total of four machine learning models, including LambdaMART, XGBoost, random forest, and SVM, were trained through 5-fold grid search with cross validation. The grid search takes an exhaustive search strategy over all combinations of pre-specified parameter values. All models were trained on a high-performance-computing platform with a 60GB V100 graphical processing unit (GPU). The parameters were finetuned and determined with the best performance on the training set. For comparison, the performance of FPocket is reported, in which the pocket with the highest score was treated as the positive (allosteric) prediction and others as negative (non-allosteric) predictions based on FPocket results. Similarly for the LambdaMART predictions, the pocket with the highest prediction score in each protein was labeled as positive. This explains that precision and recall metrics have the same number in LambdaMART and FPocket models, respectively, as there is only one positive prediction. If this positive prediction is wrong, we have a false positive, and there will also be a false negative, leading to the same number of FP and FN and thus the same value of precision and recall as seen in Equation 4 and 5.

All models were evaluated using the testing set. The results are listed in Table 1. The percentage of true allosteric sites that appeared in the predicted top 1, 2, and 3 positions was calculated and abbreviated as Top 1, 2, and 3, respectively. The performance of four machine learning models was compared with FPocket. It is shown that both LambdaMART and XGBoost exhibited better performance than FPocket under all or most metrics. RF and SVM were comparable to FPocket with higher F1 scores, MCC, and Top 3 percent. Specifically, LambdaMART achieved the best performance in 8 out of 9 metrics among all models.

These models were further evaluated using the CASBench dataset. The CASBench training data was prepared with the same procedures as the ASD training data. In addition, the proteins included in the ASD training data were excluded in the CASBench set to ensure the evaluation quality. The same metrics were calculated, and the results are listed in Table 2. Compared with the numbers reported in Table 1, the performance of all models was decreased but within an acceptable range. Overall, LambdaMART is superior to FPocket and leads in 7 out of 9 metrics. Therefore, this demonstrates the ability of LambdaMART to rank protein pockets in terms of the relevance to allostery, which leads to a high F1 score, MCC, and Top 3 percent.

**Figure 2.**
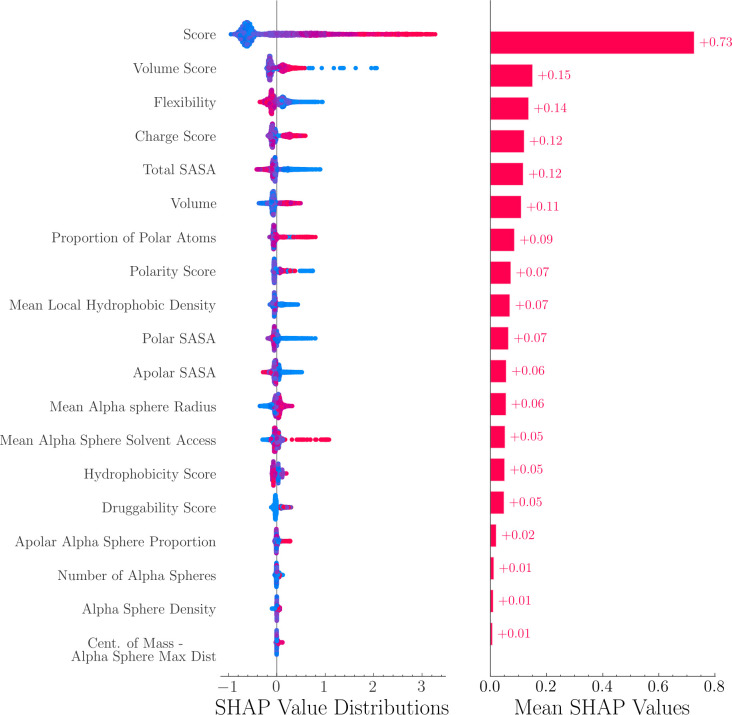
SHAP value distributions and mean values of 19 features. These features are calculated from FPocket. Red and blue colors indicate positive and negative samples, respectively. FPocket score was identified as the most important feature.

The feature importance of the LambdaMART model was analyzed using SHAP values. As shown in Figure 2, the SHAP value distributions and mean SHAP values were displayed in descending order. Figure 2 shows the distribution and mean SHAP values of the features in descending order. The results indicate that the FPocket score was the most important feature and significantly outperformed all other features. This highlights the effectiveness of the FPocket score in differentiating between allosteric and non-allosteric sites. Other features that were found to be important include the volume score, flexibility, charge score, and total solvent-accessible surface area (SASA). As seen from the SHAP value distribution, allosteric sites (represented in red) tend to have high FPocket scores, high volume scores, high charge scores, but low flexibility and low total SASA.

**Figure 3.**
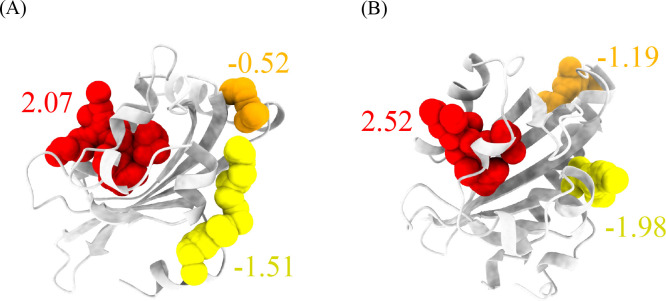
Predictions of the light-oxygen-voltage domains from (A) *Phaeodactylum tricornutum* Aureochrome 1a and (B) *Avena Sativa* phototropin 1. The top 3 pockets are highlighted in red, orange, and yellow colors. The top 1 pockets from both examples are the actual allosteric sites.

The trained LambdaMART model has been made accessible to the public through the PASSer platform (https://passer.smu.edu). Users can access the model either through the webpage or through the command line interface using the PASSer API (https://passer.smu.edu/apis/). To demonstrate the efficacy of the model, two examples that were not part of the training and validation sets are presented in Figure 3. These examples show the predicted allosteric sites of the light-oxygen-voltage domains of *Phaeodactylum tricornutum* Aureochrome 1a^34^ and *Avena Sativa* phototropin 1^35^ obtained using the LambdaMART model. The top three pockets are highlighted in red, orange, and yellow, with the corresponding predicted relevance scores, and the actual allosteric sites are highlighted in red.

## 4 Discussion

The collection and cleaning of training data is a crucial step in the development of a high-performing machine learning model. The study by Huang *et al.*^12^ applied three rules to select protein structures from the ASD dataset and curated a training set of 90 proteins, but there is no available script to automate this process. This can result in an unequal comparison between models trained on different datasets. To address this, an open-source script for protein-modulator preparation is provided with a customizable labeling strategy and sequence identity threshold. This pipeline offers a simple and customizable benchmark for evaluating various machine learning models. However, it is worth noting that proteins with multiple modulators in the same chain are discarded, as this can result in inconsistent ratios of allosteric and non-allosteric sites in each protein. Further refinement of the data cleaning process can lead to higher-quality training data.

Efforts have been invested in developing a universal model for allosteric site prediction by learning pockets from different proteins without considering the protein itself, such that all detected pockets from proteins in the training set are gathered and shuffled in a pool for training purposes. This approach, however, poses a challenge in model design and requires a model to learn a general rule that applies to all proteins of various families. Additionally, this training process is not reflective of real-world applications, where all pockets in a target protein need to be compared to determine the most probable ones. In light of these challenges, we offer a new perspective by focusing on protein-level learning to rank pockets in each protein. The model focuses on the protein level and learns a ranking pattern among pockets. The proposed LambdaMART model outperforms other popular machine learning models such as XGBoost and SVM, with high F1 score and MCC, and is capable of ranking actual allosteric sites at the top positions. This demonstrates that it is more effective to learn the relative differences among pockets rather than a universal law that applies to all proteins.

In the context of allosteric site prediction, explainable machine learning is important as it helps researchers understand how a model arrived at its predictions. This information can be useful in drug design, as it can provide insight into the factors that influence whether a pocket is likely to be an allosteric site. Tree-based models, such as random forest and gradient boosting decision tree, have good explainability as they can use metrics like Gini impurity to determine feature importance. SHAP values, a method from cooperative game theory, can also be used to quantify the contribution of each feature to the predictions made by a machine learning model. In this study, the LambdaMART model was used and its SHAP values indicated that the FPocket score was the most crucial feature, which aligns with the good performance of FPocket as a benchmark model. The SHAP values also revealed that the model tends to predict pockets with high charge, volume, and low flexibility as allosteric sites, which can benefit the development of allosteric drugs.

## 5 Conclusion

The prediction of allosteric sites is crucial to the development of allosteric drugs. While many efforts have been dedicated to constructing a universal model for such prediction, this study presents a novel approach by utilizing a relative ranking model through the learning to rank method. The proposed model outperforms other machine learning models based on various performance metrics, including a high rate of ranking true allosteric sites at the top positions. Furthermore, a customizable pipeline has been provided for the preparation of high-quality proteins for training purposes. The trained model has been deployed on the PASSer platform (https://passer.smu.edu) and is readily available for use by the scientific community.

## Figures and Tables

**Table 1.** Performance comparison of machine learning models on the ASD dataset.

Metric	LambdaMART	XGBoost	RF	SVM	FPocket

Precision	**0.662** ↑	0.586 ↑	0.528 ↓	0.444 ↓	0.556
Accuracy	**0.968** ↑	0.961 ↑	0.956 ↓	0.944 ↓	0.958
Recall	0.662 ↑	0.609 ↑	0.677 ↑	**0.758** ↑	0.556
Specificity	**0.983** ↑	0.979 ↑	0.970 ↓	0.953 ↓	0.978
F1 score	**0.662** ↑	0.596 ↑	0.593 ↑	0.559 ↑	0.556
MCC	**0.645** ↑	0.577 ↑	0.575 ↑	0.554 ↑	0.536

Top 1	**59.5%** ↑	56.6% ↑	58.0% ↑	57.5% ↑	55.6%
Top 2	**73.9%** ↑	69.6% ↓	71.0% ↓	69.6% ↓	71.5%
Top 3	**83.6%** ↑	80.7% ↑	79.7% ↑	78.3% ↑	76.8%

**Table 2.** Performance comparison of machine learning models on the CASBench dataset.

Metric	LambdaMART	XGBoost	RF	SVM	FPocket

Precision	**0.608** ↑	0.504 ↓	0.431 ↓	0.395 ↓	0.550
Accuracy	**0.963** ↑	0.953 ↓	0.941 ↓	0.932 ↓	0.956
Recall	0.608 ↑	0.657 ↑	0.767 ↑	**0.803** ↑	0.550
Specificity	**0.980** ↑	0.968 ↓	0.950 ↓	0.939 ↓	0.977
F1 score	**0.608** ↑	0.569 ↑	0.551 ↑	0.529 ↓	0.550
MCC	**0.589** ↑	0.551 ↑	0.548 ↑	0.534 ↑	0.527

Top 1	56.3% ↑	52.5% ↓	44.1% ↓	**57.3%** ↑	55.5%
Top 2	**73.7%** ↑	70.0% ↓	68.4% ↓	73.2% ↑	71.4%
Top 3	80.5% ↑	77.0% ↑	76.6% ↓	76.0% ↓	76.7%

## Data Availability

The authors declare that all data supporting the findings of this study are available within the paper.

## References

[1] Liu J.; Nussinov R. Allostery: an overview of its history, concepts, methods, and applications. PLoS computational biology 2016, 12, e1004966. 27253437 10.1371/journal.pcbi.1004966 PMC4890769

[2] Wodak S. J.; Paci E.; Dokholyan N. V.; Berezovsky I. N.; Horovitz A.; Li J.; Hilser V. J.; Bahar I.; Karanicolas J.; Stock G., Allostery in its many disguises: from theory to applications. Structure 2019, 27, 566–578. 30744993 10.1016/j.str.2019.01.003 PMC6688844

[3] Krishnan K.; Tian H.; Tao P.; Verkhivker G. M. Probing conformational landscapes and mechanisms of allosteric communication in the functional states of the ABL kinase domain using multiscale simulations and network-based mutational profiling of allosteric residue potentials. The Journal of Chemical Physics 2022, 157, 245101. 36586979 10.1063/5.0133826 PMC11184971

[4] Fenton A. W. Allostery: an illustrated definition for the ‘second secret of life’. Trends in biochemical sciences 2008, 33, 420–425. 18706817 10.1016/j.tibs.2008.05.009 PMC2574622

[5] Nussinov R.; Tsai C.-J. Allostery in disease and in drug discovery. Cell 2013, 153, 293–305. 23582321 10.1016/j.cell.2013.03.034

[6] Peracchi A.; Mozzarelli A. Exploring and exploiting allostery: Models, evolution, and drug targeting. Biochimica et Biophysica Acta (BBA)-Proteins and Proteomics 2011, 1814, 922–933. 21035570 10.1016/j.bbapap.2010.10.008

[7] Wu N.; Strömich L.; Yaliraki S. N. Prediction of allosteric sites and signaling: Insights from benchmarking datasets. Patterns 2022, 3, 100408. 35079717 10.1016/j.patter.2021.100408 PMC8767309

[8] Christopoulos A.; May L.; Avlani V. A.; Sexton P. M. G-protein-coupled receptor allosterism: the promise and the problem (s). Biochemical Society Transactions 2004, 32, 873–877. 15494038 10.1042/BST0320873

[9] De Smet F.; Christopoulos A.; Carmeliet P. Allosteric targeting of receptor tyrosine kinases. Nature biotechnology 2014, 32, 1113–1120. 10.1038/nbt.3028 25380447

[10] Lu S.; Huang W.; Zhang J. Recent computational advances in the identification of allosteric sites in proteins. Drug discovery today 2014, 19, 1595–1600. 25107670 10.1016/j.drudis.2014.07.012

[11] Lu S.; Shen Q.; Zhang J. Allosteric methods and their applications: facilitating the discovery of allosteric drugs and the investigation of allosteric mechanisms. Accounts of Chemical Research 2019, 52, 492–500. 30688063 10.1021/acs.accounts.8b00570

[12] Huang W.; Lu S.; Huang Z.; Liu X.; Mou L.; Luo Y.; Zhao Y.; Liu Y.; Chen Z.; Hou T., Allosite: a method for predicting allosteric sites. Bioinformatics 2013, 29, 2357–2359. 23842804 10.1093/bioinformatics/btt399

[13] Chen A. S.-Y.; Westwood N. J.; Brear P.; Rogers G. W.; Mavridis L.; Mitchell J. B. A random forest model for predicting allosteric and functional sites on proteins. Molecular informatics 2016, 35, 125–135. 27491922 10.1002/minf.201500108

[14] Tian H.; Jiang X.; Tao P. PASSer: prediction of allosteric sites server. Machine learning: science and technology 2021, 2, 035015. 34396127 10.1088/2632-2153/abe6d6 PMC8360383

[15] Xiao S.; Tian H.; Tao P. PASSer2. 0: Accurate Prediction of Protein Allosteric Sites Through Automated Machine Learning. Frontiers in Molecular Biosciences 2022, 9, 879251. 35898310 10.3389/fmolb.2022.879251 PMC9309527

[16] Chen T.; Guestrin C. Xgboost: A scalable tree boosting system. Proceedings of the 22nd acm sigkdd international conference on knowledge discovery and data mining. 2016; pp 785–794.

[17] Kipf T. N.; Welling M. Semi-supervised classification with graph convolutional networks. arXiv preprint arXiv:1609.02907 2016,

[18] Panjkovich A.; Daura X. Exploiting protein flexibility to predict the location of allosteric sites. BMC bioinformatics 2012, 13, 1–12. 23095452 10.1186/1471-2105-13-273 PMC3562710

[19] Laine E.; Goncalves C.; Karst J. C.; Lesnard A.; Rault S.; Tang W.-J.; Malliavin T. E.; Ladant D.; Blondel A. Use of allostery to identify inhibitors of calmodulin-induced activation of Bacillus anthracis edema factor. Proceedings of the National Academy of Sciences 2010, 107, 11277–11282. 10.1073/pnas.0914611107 PMC2895076 20534570

[20] Goncearenco A.; Mitternacht S.; Yong T.; Eisenhaber B.; Eisenhaber F.; Berezovsky I. N. SPACER: server for predicting allosteric communication and effects of regulation. Nucleic acids research 2013, 41, W266–W272. 23737445 10.1093/nar/gkt460 PMC3692057

[21] Panjkovich A.; Daura X. PARS: a web server for the prediction of protein allosteric and regulatory sites. Bioinformatics 2014, 30, 1314–1315. 24413526 10.1093/bioinformatics/btu002

[22] Huang Z.; Zhu L.; Cao Y.; Wu G.; Liu X.; Chen Y.; Wang Q.; Shi T.; Zhao Y.; Wang Y., ASD: a comprehensive database of allosteric proteins and modulators. Nucleic acids research 2011, 39, D663–D669. 21051350 10.1093/nar/gkq1022 PMC3013650

[23] Huang W.; Wang G.; Shen Q.; Liu X.; Lu S.; Geng L.; Huang Z.; Zhang J. ASBench: benchmarking sets for allosteric discovery. Bioinformatics 2015, 31, 2598–2600. 25810427 10.1093/bioinformatics/btv169

[24] Zlobin A.; Suplatov D.; Kopylov K.; Svedas V. CASBench: a benchmarking set of proteins with annotated catalytic and allosteric sites in their structures. Acta Naturae 2019, 11, 74–80. 31024751 PMC6475866

[25] Trotman A. Learning to rank. Information Retrieval 2005, 8, 359–381.

[26] Ru X.; Ye X.; Sakurai T.; Zou Q. NerLTR-DTA: drug–target binding affinity prediction based on neighbor relationship and learning to rank. Bioinformatics 2022, 38, 1964–1971. 35134828 10.1093/bioinformatics/btac048

[27] Furui K.; Ohue M. Compound virtual screening by learning-to-rank with gradient boosting decision tree and enrichment-based cumulative gain. arXiv preprint arXiv:2205.02169 2022,

[28] Ke G.; Meng Q.; Finley T.; Wang T.; Chen W.; Ma W.; Ye Q.; Liu T.-Y. Lightgbm: A highly efficient gradient boosting decision tree. Advances in neural information processing systems 2017, 30.

[29] Pedregosa F.; Varoquaux G.; Gramfort A.; Michel V.; Thirion B.; Grisel O.; Blondel M.; Prettenhofer P.; Weiss R.; Dubourg V., Scikit-learn: Machine learning in Python. the Journal of machine Learning research 2011, 12, 2825–2830.

[30] Yin C.; Song Z.; Tian H.; Palzkill T.; Tao P. Unveiling the structural features that regulate carbapenem deacylation in KPC-2 through QM/MM and interpretable machine learning. Physical Chemistry Chemical Physics 2023, 25, 1349–1362. 36537692 10.1039/d2cp03724f PMC11162551

[31] Lundberg S. M.; Erion G.; Chen H.; DeGrave A.; Prutkin J. M.; Nair B.; Katz R.; Himmelfarb J.; Bansal N.; Lee S.-I. From local explanations to global understanding with explainable AI for trees. Nature machine intelligence 2020, 2, 56–67. 10.1038/s42256-019-0138-9 PMC7326367 32607472

[32] Chicco D.; Jurman G. The advantages of the Matthews correlation coefficient (MCC) over F1 score and accuracy in binary classification evaluation. BMC genomics 2020, 21, 1–13. 10.1186/s12864-019-6413-7 PMC6941312 31898477

[33] Burley S. K.; Berman H. M.; Kleywegt G. J.; Markley J. L.; Nakamura H.; Velankar S. Protein Data Bank (PDB): the single global macromolecular structure archive. Protein Crystallography 2017, 627–641. 10.1007/978-1-4939-7000-1_26 PMC5823500 28573592

[34] Tian H.; Trozzi F.; Zoltowski B. D.; Tao P. Deciphering the allosteric process of the Phaeodactylum tricornutum Aureochrome 1a LOV domain. The Journal of Physical Chemistry B 2020, 124, 8960–8972. 32970438 10.1021/acs.jpcb.0c05842 PMC8216836

[35] Ibrahim M. T.; Trozzi F.; Tao P. Dynamics of hydrogen bonds in the secondary structures of allosteric protein Avena Sativa phototropin 1. Computational and structural biotechnology journal 2022, 20, 50–64. 34976311 10.1016/j.csbj.2021.11.038 PMC8671528

